# Intracellular Oxidant Activity, Antioxidant Enzyme Defense System, and Cell Senescence in Fibroblasts with Trisomy 21

**DOI:** 10.1155/2015/509241

**Published:** 2015-03-17

**Authors:** Víctor Rodríguez-Sureda, Ángel Vilches, Olga Sánchez, Laura Audí, Carmen Domínguez

**Affiliations:** ^1^Center for Biomedical Network Research on Rare Diseases (CIBERER), 08028 Barcelona, Spain; ^2^Biochemistry and Molecular Biology Research Center for Nanomedicine, Vall d'Hebron Research Institute, 08035 Barcelona, Spain; ^3^Maternal and Child Health and Development Network II (SAMID II), Institute of Health Carlos III, 28029 Madrid, Spain; ^4^Paediatric Endocrine Service, Vall d'Hebron University Hospital, 08035 Barcelona, Spain

## Abstract

Down's syndrome (DS) is characterized by a complex phenotype associated with chronic oxidative stress and mitochondrial dysfunction. Overexpression of genes on chromosome-21 is thought to underlie the pathogenesis of the major phenotypic features of DS, such as premature aging. Using cultured fibroblasts with trisomy 21 (T21F), this study aimed to ascertain whether an imbalance exists in activities, mRNA, and protein expression of the antioxidant enzymes SOD1, SOD2, glutathione-peroxidase, and catalase during the cell replication process *in vitro*. T21F had high SOD1 expression and activity which led to an interenzymatic imbalance in the antioxidant defense system, accentuated with replicative senescence. Intracellular ROS production and oxidized protein levels were significantly higher in T21F compared with control cells; furthermore, a significant decline in intracellular ATP content was detected in T21F. Cell senescence was found to appear prematurely in DS cells as shown by SA-*β*-Gal assay and p21 assessment, though not apoptosis, as neither p53 nor the proapoptotic proteins cytochrome c and caspase 9 were altered in T21F. These novel findings would point to a deleterious role of oxidatively modified molecules in early cell senescence of T21F, thereby linking replicative and stress-induced senescence in cultured cells to premature aging in DS.

## 1. Introduction

Down's syndrome (DS) is a developmental abnormality characterized by the presence of a third (partial or total) copy of chromosome 21. Trisomy 21 is the most common genomic aneuploidy with an incidence of approximately 1 in 700 live births [1]. DS is characterized by well-defined and distinctive phenotypic features including craniofacial dysmorphism, mental retardation, almost constant muscular hypotonia, and joint laxity. Further potential abnormalities and the clinical picture include heart defects, digestive malformations, congenital cataract, short stature, sensory deficiencies, Hirschsprung's disease, West's syndrome, seizures, propensity to leukemias and other blood diseases, autoimmune and endocrine disorders, premature aging, and Alzheimer-type neuronal pathology by the third to the fourth decade of life [2–5]. Although the DS phenotype has become better known in the last fifty years, the underlying pathogenic mechanisms of this complex syndrome and how the additional chromosome 21 causes this array of diseases remain to be elucidated. Overexpression of a number of genes located in chromosome 21 has been considered to be the central point of the DS phenotype since increased synthesis of these gene products could cause an imbalance in various biochemical pathways resulting in dysregulation of physiologic networks.

Reactive oxygen species (ROS) are generated in aerobic organisms during physiologic or physiopathologic oxidative metabolism. These species are harmful if not neutralized by the antioxidant enzyme defense system. Firstly, Cu/Zn-superoxide dismutase-1 (SOD1, EC 1.51.1.1), a major cytoplasmic antioxidant enzyme, catalyzes the dismutation of superoxide radicals to molecular oxygen and hydrogen peroxide which, in turn, is converted by either glutathione peroxidase (GPx, EC 1.11.1.9) or catalase (CAT, EC 1.11.1.6) to water and oxygen, thus providing a combined enzymatic action against oxygen toxicity. An imbalance in the ratio of SOD to GPx and CAT results in the accumulation of H_2_O_2_ which may participate in the Fenton reaction, resulting in the formation of noxious hydroxyl radicals. As the gene coding for the enzyme SOD1 is localized to 21q22.1, almost all tissues of DS patients, various trisomy 21 cultured cells [6], and the brains of DS patients [7] overexpress this gene. Overexpression of SOD1, as a consequence of gene dosage in trisomy 21 cells, can lead to increased oxidative stress (OS) caused by an altered SOD/(GPx+CAT) activity ratio that may upset the oxidant-antioxidant balance, thereby resulting in oxidative damage to molecules and vital cellular targets. In fact, evidence for a multiple prooxidant state in young DS patients would appear to support the hypothesis that ROS overgeneration could potentially contribute to some pathologic manifestations of DS, especially accelerated aging [8, 9]. However, this hypothesis has never been unequivocally tested since cells overexpressing SOD1 have been shown to produce less H_2_O_2_ [10] which would lend support to the hypothesis proposed by Liochev and Fridovich [11] that more efficient superoxide anion dismutation in DS would prevent high H_2_O_2_ levels since excess SOD1 can catalyze surrogate reactions, thereby generating hydroxyl radicals from H_2_O_2_. Furthermore, transgenic mice designed to overexpress SOD1 turned out to be even more resistant to oxygen toxicity than controls [12]. Another study showed that SOD1 overexpression increased the survival of transplanted neurons [13].

The net intracellular oxidant activity depends on the overall balance between ROS generation and the capacity of cells to buffer these highly reactive species; however, when this capacity is exceeded, oxidative stress is produced and, consequently, ROS cause damage to macromolecules such as protein, lipids, and DNA. Oxidative stress is implicated in the pathogenesis of diverse chronic or acute diseases as well as in aging [14] and has been associated with DS and its major phenotypic features such as premature aging [15].

Normal human fibroblasts cultured* in vitro* have limited proliferation potential and eventually become senescent as a result of serial passages, commonly known as replicative senescence giving rise to the idea that cellular senescence might be a determinant of human aging.* In vitro* cellular senescence is thus regarded as a useful model for elucidating molecular mechanisms that underlie organismal aging; furthermore, replicative cell senescence is continually being shown to represent a valid model of* in vivo* aging [16]. In addition to replicative senescence, cellular senescence can also be induced by various forms of stress including oxidative stress; thus, cultured fibroblasts with trisomy 21 can serve as an excellent* in vitro* tool for aging research since they can become oxidative stress-induced senescent cells in which, furthermore, replicative senescence can be induced.

The hypothesis of this study was that fibroblasts with trisomy 21 (T21F) have high SOD expression and activity which lead to an imbalance in the antioxidant enzymatic systems, thereby provoking overgeneration of ROS and cellular oxidative damage that could pathophysiologically underlie premature cell senescence in DS. In addition to antioxidant enzyme activity alterations, mitochondrial dysfunction [17], previously reported in DS, would affect cell energy production and increase intracellular oxidant activity, which combined could accelerate cellular senescence in T21F.

This work aimed to assess intracellular oxidant activity of cultured T21F and ascertain whether an imbalance exists in the activities, mRNA and protein expression of antioxidant enzymes SOD1, SOD2, GPx, and CAT at low (3–7) and high passages (8–12) during the cell replication process* in vitro*. In an attempt to determine whether oxidative stress is related to premature senescence in T21F, senescence-associated *β*-galactosidase activity (SA-*β*-Gal) and the expression of proteins p53 and p21 were analyzed at low and high passages during* in vitro* cell replication. To our knowledge, this is the first study relating, ROS generation, reduced ATP levels, oxidative stress-induced molecular damage, and cellular replicative senescence in fibroblasts from DS; the results of this study shed new light on the complex relationship between oxidative stress and premature senescence in DS.

## 2. Materials and Methods

### 2.1. Reagents and Materials

Chemicals were of analytical grade and purchased from Sigma (St. Louis, MO, USA) unless otherwise stated: 2,2′-azobis-2-methyl-propanimidamide dihydrochloride (AAPH), 1-butanol, 3,5-di-*tert*-4-butylhydroxytoluene, 4-methylumbelliferone (4-MU), 4-MU-*β*-D-galactopyranoside, 4-MU-*β*-D-glucuronide, 1,1,3,3-tetraethoxypropane, 2-thiobarbituric acid, adenosine 5′-triphosphate assay mix, acetonitrile, diethylenetriaminepentaacetic acid (DTPA), dimethyl sulphoxide (DMSO), Dulbecco's Modified Eagle Medium (DMEM), Eagle's Minimal Essential Medium (MEM), fetal calf serum (FCS), Hank's buffered salt solution (HBSS), sodium chloride, sodium dodecyl sulfate (SDS), somatic cell ATP releasing reagent,* tert*-butyl hydroperoxide (TBHP), Tris·HCl, Triton X-100, and Tween-20.

Penicillin, streptomycin, 5-(and-6-)-chloromethyl-2′,7′-dichlorodihydrofluorescein diacetate acetyl ester probe (CM-H_2_DCFDA), MitoSOX Red Mitochondrial Superoxide Indicator, MitoTracker Green FM, and Hoechst 33342 were purchased from Invitrogen (Carlsbad, CA, USA). Cell Proliferation Kit II (XTT) was purchased from Roche Applied Science (Basel, Switzerland). 96-well and 12-well clear-bottom culture plates were from Corning (Corning, NY, USA). SOD activity determination kit (RANSOD) was from Randox (Crumlin, UK) and GPx activity determination kit (cGPx-340) was from Bioxytech (Deltaclon, Madrid, Spain). The following antibodies were used for immunoblotting: SOD1 (sc-30080) and SOD2 (sc-11407) were purchased from Santa Cruz Biotechnology (Santa Cruz, CA, USA). Dinitrophenyl-KLH (A-6430) was from Invitrogen. p21 Waf1/Cip 1 (#2947), cytochrome c (#4272), and cleaved caspase 9 (#9505) were from Cell Signaling (Danvers, MA, USA). p53 (P8999), *β*-actin (A3854), rabbit IgG-HRP (A0545), and mouse IgG-HRP (A2304) were from Sigma-Aldrich. Mammalian protein extraction reagent (M-PER), Halt Protease Inhibitor Cocktail, and SuperSignal West Pico were from Pierce Biotechnology (Rockford, IL, USA). Immobilon-P was purchased from Millipore Corporation (Billerica, MA, USA). RNeasy Mini Kit and RNase-Free DNase Set were from Qiagen (Hilden, Germany). RevertAid H minus first strand cDNA synthesis kit was from Fermentas (St. Leon-Rot, Germany). TaqMan Gene Expression Assays (predesigned probe and primer sets) were from Applied Biosystems (Foster City, CA, USA) and senescence detection kit was from BioVision (Mountain View, CA, USA). Human p21 Waf1/Cip1 and Human p 53 ELISA kits were from Cell Signaling (Danvers, MA, USA) and Biovendor (Brno, Czech Republic), respectively.

### 2.2. Human Dermal Fibroblast Cultures from Down's Syndrome and Control Fetuses

Abdominal skin biopsies from 10 human fetuses at 9–22 weeks of gestation, products of legal terminations, were collected with informed parental consent within 12 h postmortem from the Fetal Tissue Bank at the Hospital Universitari Vall d'Hebron (fetaltissuepath@vhebron.net). Five fetuses were confirmed as having Down's syndrome: three 47,XY + 21 and two 47,XX + 21. The five remaining fetuses, two diagnosed of complex cardiac malformation (46,XX), one anhydramnios with previous membrane rupture (46,XY) and two occipital encephalocele (46,XX and 46,XY), were used as controls (CF).

Primary fibroblasts were obtained from skin explants in a 25 cm^2^ culture flask with Eagle's Minimal Essential Medium (MEM) supplemented with nonessential amino acids, 10% fetal calf serum (FCS), and antibiotics (100 IU/mL penicillin and 100 mg/mL streptomycin). The flasks were maintained at 37°C in a 95% humid air 5% CO_2_ atmosphere. Fibroblasts were released by enzymatic digestion with trypsin and subcultured in a 75 cm^2^ plastic culture flask with 12 mL of medium. Cultures were fed by changing the medium every 3 days. After reaching confluence, cells were subjected to different passages and fold increase in the cell count during each passage was calculated or washed and the pellet resuspended in 1 mL MEM with 10% FCS and DMSO and frozen (24 h at −80°C and then in liquid nitrogen). Cells were thawed and reincorporated into the cultures for ensuing experiments at different passages for which fibroblasts were grown in medium supplemented with 10% FCS. We observed that cultured FT21 aged more prematurely than control fibroblasts since, beyond passage 11, they showed progressive morphologic characteristics typical of senescent cells (enlarged vesicle-rich cytoplasm, diminished cell density, and increased times to reach confluence), which rendered their culture inviable beyond passage 13. Therefore, the following population doubling (PD) cut-offs were used to differentiate cell aging in two stages: nonsenescent cells (8–16 PD, low passages (LP)) and aged fibroblasts (24–32 PD, high passages (HP)). Cells were seeded at all times in 96-well culture microplates and incubated for 48 hours to allow them to attach to the plates.

### 2.3. Prooxidant Challenge

Cells were grown in 96-well culture plates at 10^4^ cells/well for ROS assessment and 5 · 10^3^ cells/well for ATP and cell viability. On the day of the experiment, the culture medium was removed and the cells were washed twice with 100 *μ*L of Hank's buffered salt solution (HBSS, Sigma). Cells were then incubated for 1 hour at 37°C with either 100 *μ*L of HBSS (untreated cells), 100 *μ*L of 10 mM AAPH in HBSS (AAPH-treated cells), or 100 *μ*L of 100 *μ*M TBHP in HBSS (TBHP-treated cells), washed as above and processed for the determination of cell viability, ATP and ROS (see below).

### 2.4. Cell Viability

Cell viability was assessed with the Cell Proliferation Kit II (XTT). In brief, cells grown on 96-well culture plates were washed twice with 100 *μ*L of HBSS to remove culture medium. Then, 100 *μ*L of HBSS and 50 *μ*L of the reaction mixture were added to each well. Plates were incubated for 4 hours at 37°C and absorbance was read at 460 nm with a reference filter of 680 nm.

### 2.5. Cell ATP Content

Intracellular ATP determination was performed by a bioluminescence assay based on the ATP-dependent luciferin-luciferase reaction [18]. A new internal calibration standard of ATP was prepared each day in a range from 1 to 100 *μ*g ATP/mL prior to readings. To determine the cellular ATP content, cells grown on 96-well white clear-bottom plates were first incubated for 3 minutes with 25 *μ*L somatic cell ATP releasing reagent and then for 3 minutes with 25 *μ*L of sterile water. The plates were placed in a Luminoskan RS luminometer (Thermo Fisher Scientific, Waltham, MA, USA), and 50 *μ*L of the luciferase-containing buffer (adenosine 5′-triphosphate assay mix) was added to each well just before measurement of the light emitted, which is proportional to the ATP concentration. As the different batches of luciferase yielded varying linear responses, reading values in each microplate were converted into percentages, with the control values of CF under the same conditions (untreated, AAPH and TBHP) considered to be 100%.

### 2.6. Measurement of Reactive Oxygen Species

Intracellular ROS levels were assessed using the CM-H_2_DCFDA probe. Cells grown in 96-well flat clear-bottom black polystyrene microplates were washed twice with 150 *μ*L of HBSS to remove culture medium. Then, 50 *μ*L of the probe (7.21 *μ*M in HBSS) was added to each well and the plates were incubated for 30 minutes at 37°C. Excess probe was discarded and 100 *μ*L of the prooxidant solutions prepared in HBSS was added (see above). After the incubation, cells were lysed by adding 50 *μ*L of 0.5% Triton X-100 in PBS. The plates were left protected from light for 3 minutes in a shaker and fluorescence was read at 538 nm with an excitation filter of 485 nm. Cellular levels of ROS were also visualized under microscope using CM-H_2_DCFDA fluorescence live cell imaging; cell fluorescence images were captured using an epifluorescence microscope, equipped with a digital camera and image processing software (NIS-Elements, Nikon Instruments Europe, Amstelveen, Netherlands).

### 2.7. Mitochondrial Superoxide Generation

Mitochondrial superoxide levels were assessed using the MitoSOX probe. Cells grown in 24-well flat clear-bottom polystyrene microplates were washed twice with 1 mL of prewarmed HBSS to remove culture medium. Then, 1 mL of the probe (5 *μ*M in HBSS) was added to each well, and the plates were incubated for 10 minutes at 37°C in a 5% CO_2_ atmosphere protected from light. After this period, cells were washed and images were taken using a Nikon Eclipse Ti epifluorescence microscope using a TRITC filter set.

### 2.8. Enzyme Activities

Total SOD activity was assayed using the RANSOD kit, with minor modifications. In brief, fibroblasts were lysed in SOD buffer (500 mM Tris·HCl; 10 mM DTPA; pH 8.2) by sonication at 4°C and centrifuged for 10 min at 10,000 g. SOD activity was assayed in 15 *μ*L of the supernatant and the results are expressed as U/mg protein (one unit of SOD is that which causes 50% of the INT reduction rate under the assay conditions).

GPx activity was assayed using the cGPx-340 kit, with minor modifications. In brief, fibroblasts were lysed in GPx buffer (50 mM Tris·HCl; 5 mM EDTA; 1 mM DTT; pH 7.5) by sonication at 4°C and centrifuged for 10 min at 10,000 g. GPx activity was assayed in 70 *μ*L of the supernatant using an undiluted sample and two samples diluted at 1/2 and 1/4 and the results are expressed as mU/mg protein (one unit of GPx is that which produces 1 *μ*mol of NADP^+^ per minute at pH 8.0 and 25°C, in a coupled reaction with reduced glutathione, glutathione reductase, and* tert*-butyl hydroperoxide).

Catalase was assayed by the Aebi method [19]. In brief, fibroblasts were lysed in CAT buffer (200 mM KH_2_PO_4_/K_2_HPO_4_; pH 7) by sonication at 4°C and centrifuged for 10 min at 10,000 g. CAT activity was assayed in 20 *μ*L of the supernatant by adding 1 mL of 20 mM H_2_O_2_ in CAT buffer. Absorbance of the samples was then recorded for 45 seconds and the rate of NADPH consumption per minute was calculated. Results were expressed as U/mg protein (one unit of CAT is that which decomposes 1 *μ*mol of H_2_O_2_ per minute at pH 7.0 and 25°C).

*β*-glucuronidase (*β*-Gluc, EC 3.2.1.31) and *β*-galactosidase (*β*-Gal, EC 3.2.1.23) were determined using the fluorogenic substrates 4-methylumbelliferyl-*β*-D-glucuronide (3.46 mM in 40 mM acetic-acetate buffer, pH 3.5) and 4-methylumbelliferyl-*β*-D-galactopyranoside (2 mM in 0.1 M citrate-phosphate buffer adjusted to pH 4 or 6; 0.2 M NaCl), respectively. Fibroblasts were lysed by sonication at 4°C in distilled water. 25 *μ*L of each sample (diluted 1/10 in water) was incubated with 100 *μ*L of the substrate at 37°C for 30 minutes. The reaction was then stopped by adding 1.25 mL of 100 mM glycine-NaOH buffer (pH 10.4) and the fluorescence was read in a F-2500 FL spectrophotometer (excitation: 365 nm; emission: 450 nm). After blank subtraction, activity was calculated with respect to a standard curve of 4-methylumbelliferone and expressed in nmol per minute and mg of protein in the lysate. Protein content was determined according to the method of Lowry [20] adapted to the sample characteristics, or with the BCA assay [21].

### 2.9. Western Blot

Western blotting was performed using antibodies against SOD1 (1/5,000), SOD2 (1/2,000), p21 (1 : 1,000), p53 (1 : 2,500), cytochrome c (1 : 1,000), cleaved caspase 9 (1 : 1,000), and *β*-actin (1/400,000). Cells were lysed by sonication in M-PER containing protease inhibitors, and 20 *μ*g of protein was applied to each well. Samples were separated on 15% SDS-polyacrylamide gels and then transferred onto PVDF membranes. Blots were blocked for 1 hour at 37°C in TBS (10 mM Tris·HCl, 150 mM NaCl, pH 7.4) with 5% powdered milk and then incubated overnight at 4°C with the primary antibodies (1% powdered milk and 0.01% (v/v) Tween 20 in TBS). Blots were washed three times in TBS-Tween (0.05% (v/v) Tween-20 in TBS) and three times in TBS and then incubated with a horseradish peroxidase-conjugated secondary antibody in incubation buffer for 2 hours at room temperature. After washing, immunocomplexes were developed using an enhanced horseradish peroxidase-luminol chemiluminescence reagent (SuperSignal West Pico) according to the manufacturer's instructions.

### 2.10. Quantitative Real-Time PCR for mRNA Expression Analyses

Gene expression was analyzed with quantitative real-time PCR (1 ng cDNA for each reaction) using a Prism 7000 Sequence Detection System (Applied Biosystems). Total RNA was isolated from cultured fibroblasts (1 · 10^6^ cells/column) using the RNeasy Mini Kit and genomic DNA was removed using the RNase-Free DNase Set. RNA (200 ng for each reaction) was used for cDNA synthesis with random hexamer primers using the RevertAid H minus first strand cDNA synthesis kit. TaqMan Gene Expression Assays (predesigned probe and primer sets) were obtained from Applied Biosystems. SOD1 (assay ID Hs00166575_m1), SOD2 (assay ID Hs00167309_m1), and GPx (assay ID Hs00829989_gH) mRNA expression levels were normalized to the levels of human DNA-directed RNA-polymerase II (assay ID Hs00172187_m1) and human *β*-actin (Human ACTB Endogenous Control). Relative expression was calculated by the double delta Ct method [22], using the Sequence Detection Software (v. 1.2.3) from Applied Biosystems.

### 2.11. Lipoperoxidation (LPx) and Protein Carbonyl Groups (PGC) in Cell Lysates

Cell lysates of fibroblasts were prepared by resuspending the extracted cells in distilled water followed by sonification (10 sec, three times in ice). Cellular MDA concentration was determined as its diethylthio-barbituric acid adduct (TBA-MDA), after reverse-phase isocratic HPLC separation of the MDA-TBA complex, as previously described in detail [23, 24].

Protein carbonyl content was analyzed by Western blot analysis. Cell lysates in M-PER were denatured with SDS and incubated with dinitrophenyl-hydrazine for 5 minutes at room temperature. Samples were then neutralized and reduced by addition of 2-mercaptoethanol and loaded onto 9% polyacrylamide gels. After electroblotting, oxidized proteins were detected by an anti-dinitrophenyl-KLH antibody.

### 2.12. Detection of Senescence-Associated *β*-Galactosidase

SA-*β*-Gal expression in fibroblasts was examined cytochemically with a commercial senescence detection kit, which is based on the method described by Dimri and coworkers [25]. Briefly, cells grown on 12-well culture plates (7 · 10^3^ cells/well) were fixed with 3% formaldehyde, washed, and incubated for 16 hours at 37°C with a staining solution containing 5-bromo-4-chloro-3-indolyl-*β*-D-galactopyranoside (X-Gal). Nuclei were then counterstained for 10 minutes at 37°C with the nuclear dye Hoechst 33342 (Invitrogen) and cells observed in a Nikon Eclipse Ti epifluorescence microscope (Nikon Instruments Europe, Amstelveen, Netherlands). A minimum of five random fields per well were photographed at 100x, under both bright field and UV light, for senescent cell identification and cell counting, respectively.

### 2.13. Statistical Analysis

Results are expressed as mean ± standard error of the mean (SEM). Statistical analyses were performed using GraphPad Prism version 5.00 for Windows (GraphPad Software, San Diego, CA, USA, http://www.graphpad.com/). Student's *t*-test was performed throughout unless otherwise stated. A *P* value <0.05 was considered statistically significant. Western blotting densitometric analysis was performed using Quantity One 4.6.9 software (Bio-Rad, Madrid, Spain).

## 3. Results

### 3.1. Intracellular ROS Production by Fibroblasts with Trisomy 21 and Control Fibroblasts

To ascertain the degree of oxidative stress in cultured fibroblasts with trisomy 21 and their sensitivity against a prooxidant challenge, we determined intracellular ROS production and cell viability at low and high passages. To this end, FT21 and control cells were exposed to the prooxidant chemical substances AAPH and TBHP at the concentration that produced maximum ROS induction with minimum cytotoxicity. The results showed that fibroblast viability at low passages was not significantly affected by the prooxidant treatments; however, at high passages, T21F proved to be more sensitive to prooxidant challenge, particularly with TBHP, which significantly reduced cell viability (Table 1).

**Figure 1:**
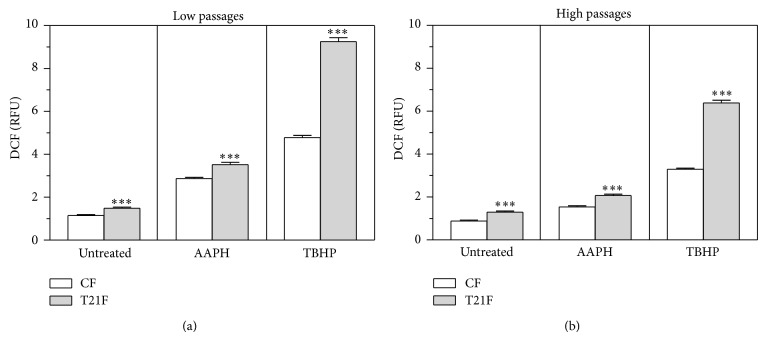
ROS production determined by dichlorofluorescein oxidative stress assay in fibroblasts with trisomy 21 (T21F) (*n* = 5) and controls (CF) (*n* = 5). Fibroblasts were grown in 96-well microplates and incubated for one hour with Hank's buffered salt solution (untreated) or supplemented with 10 mM AAPH or 100 *µ*M TBHP. ROS production was evaluated at low (a) and high (b) passages with H2DCFA-DA. Values are expressed as mean ± SEM of six separate experiments performed in quadruplicate. Statistical differences between CF and T21F were analyzed by Student's *t*-test; ^***^ *P* < 0.001.

Cells were incubated with the CM-H_2_DCFDA probe and its subsequent oxidation was quantified by measuring the fluorescence of the probe's adduct to detect ROS generation (Figure 1). Basal ROS production at low passages was found to be 30% higher in T21F (*P* < 0.001) than in control fibroblasts. ROS induction by AAPH was 23% (*P* < 0.001) greater in T21F than in CF. Both cell types, in response to TBHP, showed more intense ROS production which was far greater in T21F than in CF (*P* < 0.001) (Figure 1(a)). ROS generation in replicatively aged T21F was 48% higher than in CF (*P* < 0.001), rising significantly during replicative senescence (Figure 1(b)): from 30% in T21F cells at LP to 48% at HP (*P* < 0.01) and from 23% to 34% in AAPH-treated cells (*P* < 0.05), whereas no differences were found in TBHP-treated fibroblasts during* in vitro* aging.

**Figure 2:**
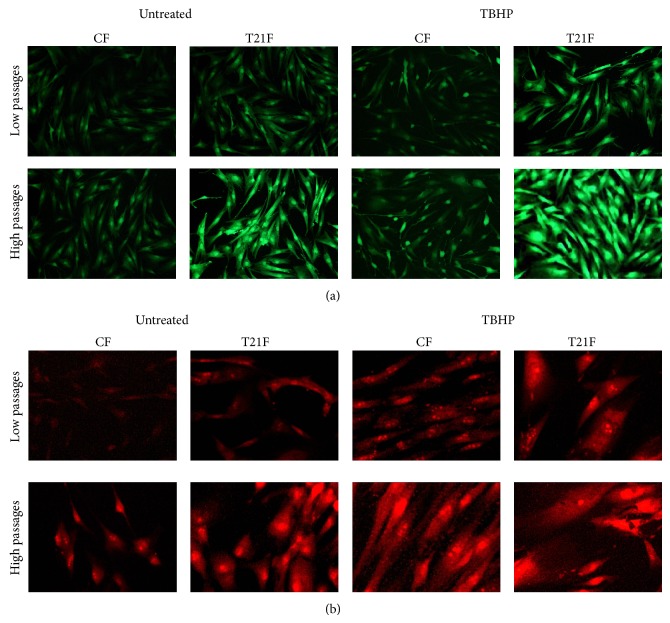
ROS and mitochondrial superoxide anion detection by fluorescence microscopy in fibroblasts with trisomy 21 (T21F) (*n* = 5) and controls (CF) (*n* = 5). Fibroblasts were grown in 96-well microplates and incubated for one hour with Hank's buffered salt solution (untreated) or supplemented with 100 *µ*M TBHP. The observed green fluorescent signal corresponds to the oxidation of the CM-H2DCFDA probe in response to intracellular ROS production in (a). The red fluorescent signal corresponds to the oxidation of the MitoSOX probe in response to superoxide radical production in the mitochondria (b). Images were taken with a fluorescence microscope at a total magnification of 200x.

ROS generation was also visualized by conventional epifluorescence microscopy in cultured fibroblasts (Figure 2(a)). Representative fluorescent microscope images showed stronger CM-H_2_DCFDA staining in T21F than in CF and the fluorescence was even more intense at high passages; the fluorescent micrographs of CM-H_2_DCFDA-loaded cells also revealed higher fluorescence intensities in cells treated with the prooxidant TBHP than in nontreated fibroblasts.

With the aim of detecting mitochondrial superoxide anion generation, the MitoSOX Red Mitochondrial Superoxide Indicator fluorescent probe was used. The probe rapidly penetrates cell mitochondria and is oxidized by superoxide anions and not by other ROS or reactive nitrogen species. The oxidized probe can be visualized by fluorescence microscopy since it emits bright red fluorescence upon binding to the nuclear DNA. Both cell types (T21F and CF) at LP and HP were treated with TBHP (microscopic images shown in Figure 2(b)). Microscopic observation of greater red fluorescence in T21F than in CF revealed higher mitochondrial superoxide anion production.

### 3.2. Intracellular ATP Levels in T21F

**Figure 3:**
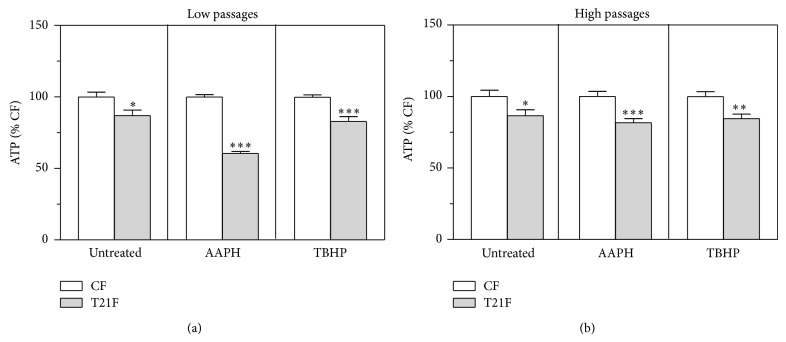
Intracellular ATP levels in fibroblasts with trisomy 21 (T21F) (*n* = 5) and controls (CF) (*n* = 5). Fibroblasts were grown in 96-well microplates and incubated for one hour with Hank's buffered salt solution (untreated) or supplemented with 10 mM AAPH or 100 *µ*M TBHP. Intracellular ATP content at low (a) and high (b) passages was expressed as a percentage of mean values obtained in the control group for each treatment. Results are expressed as mean ± SEM of six separate experiments performed in quadruplicate. Statistical differences between CF and T21F were analyzed by Student's *t*-test: ^*^ *P* < 0.05; ^**^ *P* < 0.01; ^***^ *P* < 0.001.

Levels of intracellular ATP at LP were lower in T21F fibroblasts in basal conditions (−13%, *P* < 0.05) and ATP depletion was accentuated when cells were treated with 10 mM AAPH (−40%, *P* < 0.001) or with 100 *μ*M TBHP (−17%, *P* < 0.001) (Figure 3(a)). In basal conditions, intracellular ATP content at HP was lower in T21F compared with CF (−13%, *P* < 0.05) and ATP depletion was more marked when cells were treated with either of the two prooxidants: AAPH (−18%, *P* < 0.001) or TBHP (−15%, *P* < 0.01) (Figure 3(b)).

### 3.3. Oxidative Stress Markers in T21F

**Figure 4:**
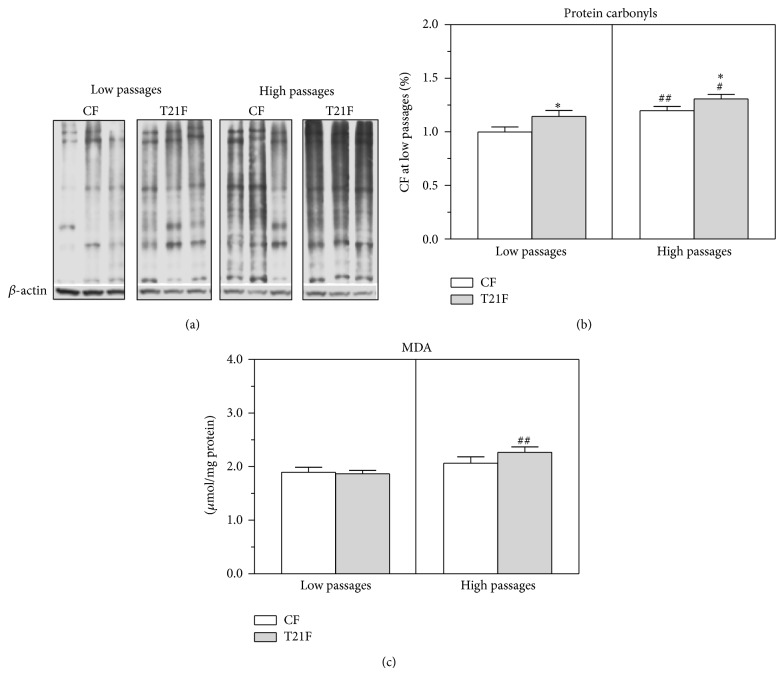
Protein carbonyls and malondialdehyde in fibroblasts with trisomy 21 (T21F) (*n* = 5) and controls (CF) (*n* = 5). (a) Representative protein carbonyl Western blot of DNPH-derivatized cell lysates in MPER. (b) Protein carbonyls were calculated from optical densities of the bands measured by an imaging technique. Results were normalized to the band intensities measured in untreated fibroblasts at low passages and expressed as mean ± SEM. (c) MDA was determined in cell lysates by HPLC. Values were corrected by the protein of the lysate and expressed as mean ± SEM. Statistical differences were analyzed by Student's *t*-test: high versus low passages (^#^ *P* < 0.05; ^##^ *P* < 0.01); T21F versus CF (^*^ *P* < 0.05).

Western blot analysis was performed to visualize oxidatively modified cellular proteins in T21F versus CF and ascertain whether these oxidized proteins varied throughout the cell aging process* in vitro* (Figure 4). Despite the variable degree of protein oxidation observed in fibroblasts from DS and control cells, T21F showed significantly more oxidized proteins than CF at both low (14%, *P* < 0.05) and high passages (9%, *P* < 0.05). Furthermore, our results showed that, throughout* in vitro* cell aging, both control (20% increase, *P* < 0.01) and T21F (14% increase, *P* < 0.05) accumulated oxidized proteins (Figures 4(a) and 4(b)). While concentrations of cellular malondialdehyde (MDA), the most representative indicator of lipid peroxidative damage, appeared to rise with* in vitro* cell aging, this increase was significant only in T21F (22%, *P* < 0.01) (Figure 4(c)).

### 3.4. Antioxidant Enzyme Activities in T21F

**Figure 5:**
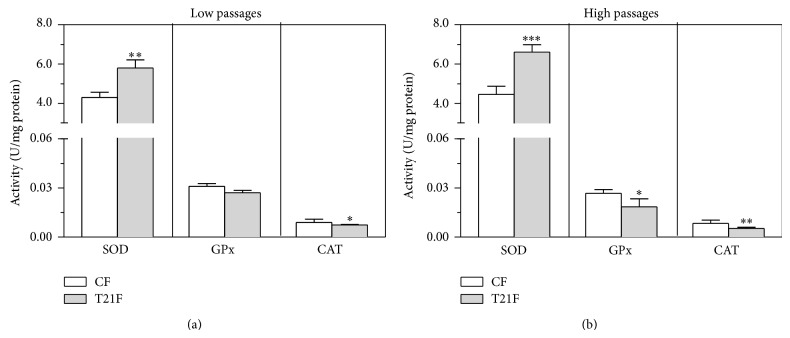
Antioxidant enzyme activities in fibroblasts with trisomy 21 (T21F) (*n* = 5) and controls (CF) (*n* = 5). Enzymatic activities in cell lysates were determined as described in Methods section. Results at low (a) and high (b) passages are expressed as mean ± SEM of 12 separate experiments performed in triplicate. Statistical differences between T21F and CF were analyzed by Student's *t*-test; ^*^ *P* < 0.05; ^**^ *P* < 0.01; ^***^ *P* < 0.001.

SOD, GPx, and CAT activities were determined to ascertain whether an imbalance existed in the antioxidant enzymatic systems between FT21 and CF and, if so, whether it persisted in successive passages. Total SOD activity from T21F cell homogenates at LP was 35% higher than in CF (*P* < 0.05) and 48% higher in fibroblasts at HP (*P* < 0.01) (Figures 5(a) and 5(b)). Intracellular GPx activity of T21F at LP was 13% lower than in the control group and 31% lower than in CF at HP (Figures 5(a) and 5(b)). Total CAT activity of T21F cell homogenates at LP was 17% lower than in CF and 38% lower than in CF at HP (Figures 5(a) and 5(b)).

The influence of replicative senescence, represented by low- and high-passage groups, on antioxidant enzyme ratios was calculated from the values of enzyme activities. The SOD/GPx ratio was 1.6-fold higher in T21F at LP than in CF, as also occurred with the SOD/CAT and SOD/(GPx+CAT) ratios. This interenzymatic activity imbalance was accentuated with replicative senescence: the SOD/GPx ratio was 2.3-fold higher in T21F than in CF at HP, the SOD/CAT ratio 2.6 times higher in T21F than in CF at HP, and the SOD/(GPx+CAT) 2.4-fold higher in T21F than in CF at HP.

### 3.5. Changes in SOD1 Expression and Activity in T21F in relation to Replicative Senescence

**Figure 6:**
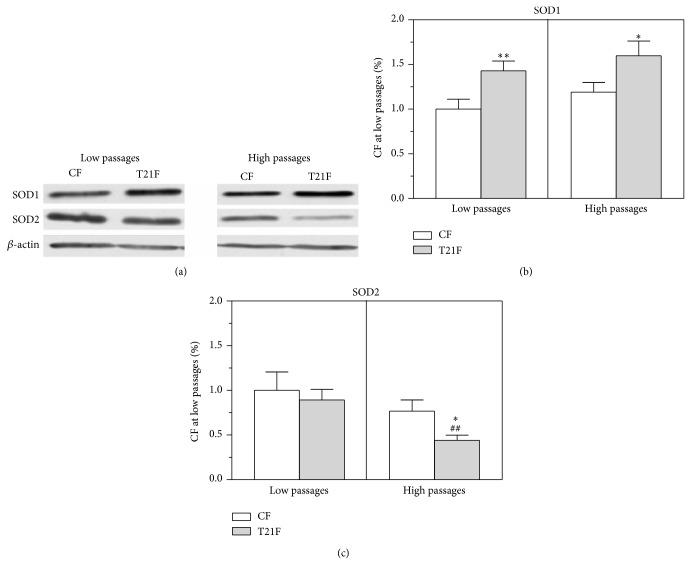
Effect of cell passages on SOD1 and SOD2 expression in fibroblasts with trisomy 21 (T21F) (*n* = 5) and controls (CF) (*n* = 5). SOD1 and SOD2 expression were analyzed by Western blot (a) and the bands were quantified by an imaging technique and normalized by *β*-actin (b and c). Results are normalized to the band intensities measured in untreated fibroblasts at low passages and expressed as mean ± SEM. Statistical differences were analyzed by Student's *t*-test: T21F versus CF (^*^ *P* < 0.05; ^**^ *P* < 0.01); high versus low passages (^##^ *P* < 0.01).

SOD1 activity (Figure 5) and mass (Figure 6) were higher in T21F, at both LP (43% mass increase, *P* < 0.01) and HP (34% mass increase, *P* < 0.05). Considering that SOD1 accounts for approximately 70–80% of total SOD activity [26], a correlation would be expected between total SOD activity and SOD1 mass as determined by Western blot. With replicative senescence, while activities of SOD in the control group were almost identical between LP and HP, its expression analysis by Western blot revealed a mass increase of 19% (*P* > 0.05) (Figures 5 and 6). In T21F, both activity and mass were slightly higher at HP (14% and 12%, resp., *P* > 0.05).

**Figure 7:**
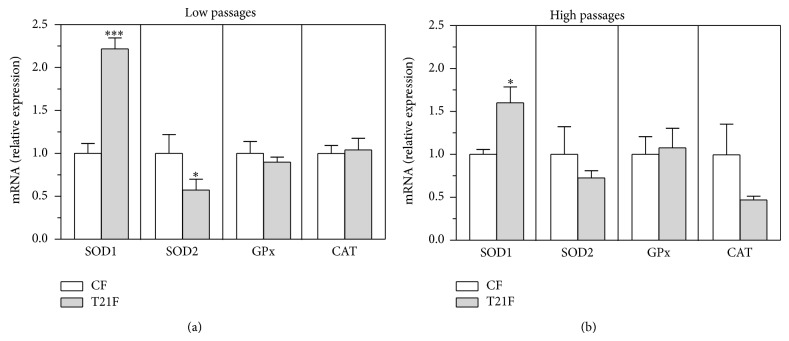
Effect of cell passages on relative mRNA expression of SOD1, SOD2, GPx, and catalase (CAT) in fibroblasts with trisomy 21 (T21F) (*n* = 5) and controls (CF) (*n* = 5). mRNA expression was analyzed by quantitative real-time PCR in fibroblasts at low and high passages. Expression data were normalized by the means of the double delta Ct method. Results at low (a) and high passages (b) are expressed as mean ± SEM. Statistical differences were analyzed by Student's *t*-test: ^*^ *P* < 0.05; ^***^ *P* < 0.001.

On the other hand, SOD2 expression was found to be decreased with replicative senescence in both control fibroblasts (23%, *P* > 0.05) and T21F (51%, *P* < 0.01) (Figure 7(c)). Moreover, at HP, SOD2 expression was significantly lower in T21F than in control fibroblasts (42% decrease, *P* < 0.05).

### 3.6. mRNA Expression of SOD1, SOD2, and GPx in T21F

Gene expression of antioxidant enzymes SOD1, SOD2, and Se-dependent GPx was analyzed by real-time quantitative PCR to ascertain whether the increased production of ROS observed at baseline in T21F was related to mRNA expression of antioxidant enzymes. The results show that the expression of SOD1 was significantly higher in T21F at both HP and LP (Figure 7); it should be noted that the differences observed between controls and T21F decreased from 122% at LP (*P* < 0.001) to 60% (*P* < 0.05) at HP. Analysis of SOD2, GPx, and CAT expression yielded no differences between groups, except for SOD2 at LP, where a significant decrease was observed in T21F (43%, *P* < 0.05). It should be noted that although not being statistically significant, CAT expression in T21F at HP was half with respect to CF.

### 3.7. Replicative Senescence in T21F

**Figure 8:**
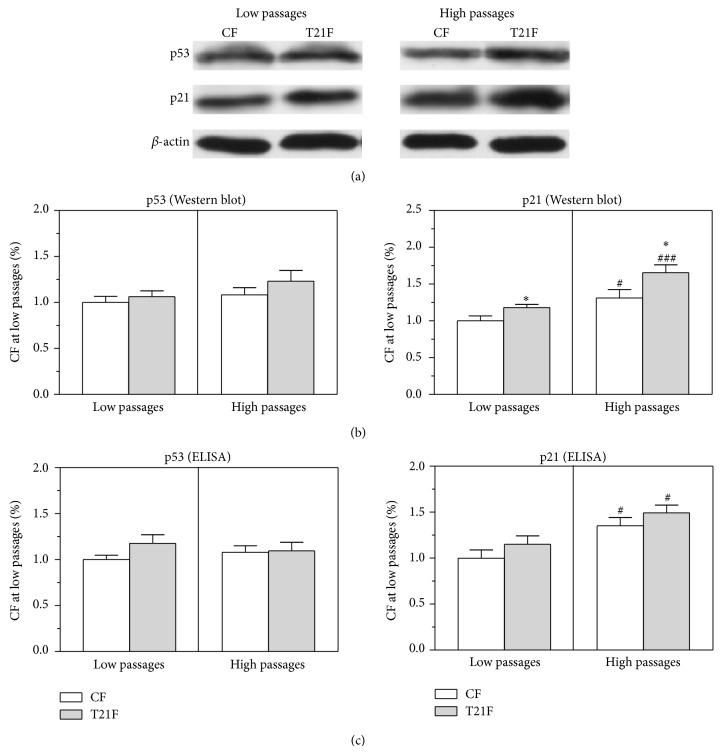
Effect of cell passages on p53 and p21 expression in fibroblasts with trisomy 21 (T21F) (*n* = 5) and controls (CF) (*n* = 5). (a) Representative Western blot of p53 and p21 in cell lysates in M-PER. (b) p53 and p21 expression was calculated from optical densities of the bands, measured by an imaging technique and normalized by *β*-actin. Results were normalized to the band intensities measured in untreated fibroblasts at low passages and expressed as mean ± SEM. (c) p53 and p21 were also quantified by ELISA in cell lysates of T21F and CF. Results were normalized to the optical densities measured in untreated fibroblasts at low passages and expressed as mean ± SEM. Statistical differences were analyzed by Student's *t*-test: T21F versus CF (^*^ *P* < 0.05) and high versus low passages (^#^ *P* < 0.05; ^###^ *P* < 0.001).

Control and T21F fibroblasts were seeded at LP and HP and expression of p21 and p53 proteins was analyzed by Western blot to ascertain whether* in vitro* replicative senescence of fibroblasts with trisomy 21 is related to premature cell aging (Figure 8). The Western blot results indicated an increase in p53 protein expression in T21F compared with controls (6% at LP and 14% at HP), whereas p21 protein expression in T21F was significantly increased at both LP (18%, *P* < 0.05) and HP (26%, *P* < 0.05) with respect to CF (Figure 8(b)).* In vitro* replicative aging led to a significant increase in p21 expression both in T21F (40% increase from EP, *P* < 0.001) and in control fibroblasts (31% increase from EP, *P* < 0.05). Results of p53 and p21 expression quantified by ELISA showed no statistically significant differences in p53 protein expression in T21F versus CF, whereas p21 protein expression significantly increased with* in vitro* replicative aging in T21F (30%, *P* < 0.05) and in control fibroblasts (35%, *P* < 0.05) (Figure 8(c)).

**Figure 9:**
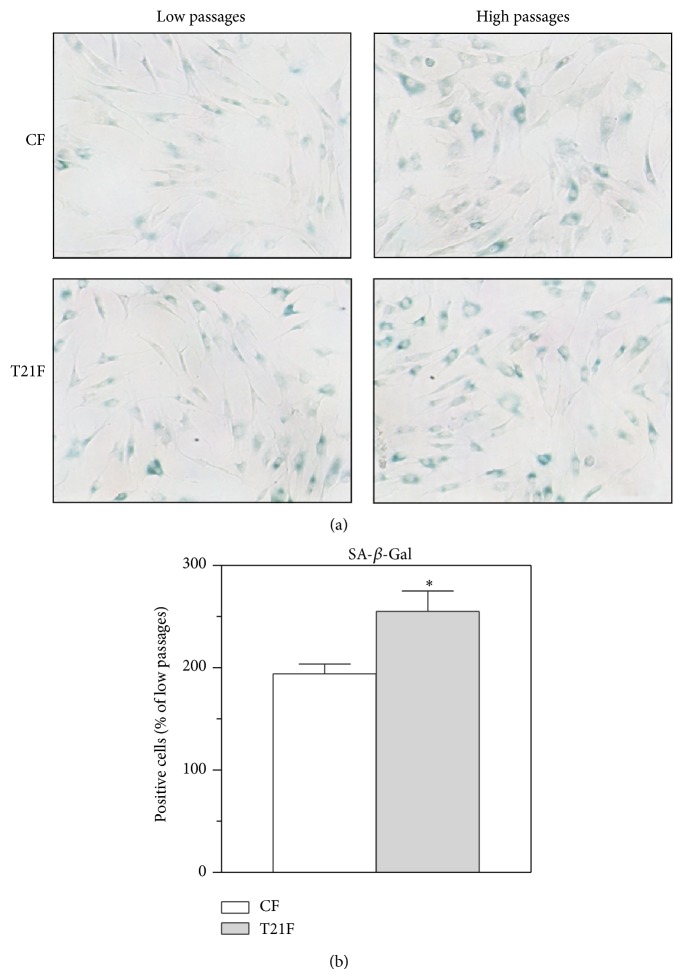
Effect of* in vitro* replicative aging on SA-*β*-Gal expression in fibroblasts with trisomy 21 (T21F) (*n* = 5) and controls (CF) (*n* = 5). (a) Representative microphotographs of X-Gal staining in T21F and CF at low and high passages. (b) Fold increase in positive cell counts for X-Gal from low to high passages in T21F and CF. Cells were seeded in 12-well plates, stained with X-Gal. A minimum of 5 random fields were photographed ×100 with phase contrast for X-Gal-positive cell count. Results are expressed as mean ± SEM. Statistical differences between the two passage groups were analyzed by Student's *t*-test: ^*^ *P* < 0.05.

Another method of cell aging assessment* in vitro* was carried out using senescence-associated *β*-galactosidase activity (Figure 9(a)). SA-*β*-Gal-positive cells were counted (minimum 5 random fields and 50–100 cells/field) and the proportions of X-Gal-positive cells at LP and HP were calculated for each cell line (Figure 9(b)). While CF underwent a twofold increase in the number of X-Gal-positive cells at HP compared with LP, X-Gal-positive T21F at HP increased 2.5 times more than at LP, which represented a 31% increase over control values (*P* < 0.05). It is noteworthy that given the great interindividual variability observed in the cell division rate and senescence process of both CF and T21F, the number of SA-Gal-positive cells was counted at low and high passages and the differences were calculated in each cell line.

To ascertain whether* in vitro* replicative aging is linked to an increase in lysosomal enzyme synthesis, *β*-galactosidase (pH 4 and 6) and *β*-glucuronidase activities were determined in T21F and CF at HP and LP (Table 2). Results indicate that in both T21F and CF replicative senescence was accompanied by varying nonsignificant increases in *β*-galactosidase pH 6 activity, whereas greater and significant increases were found in intracellular *β*-glucuronidase activity.

### 3.8. Caspase-Dependent Apoptosis in T21F

**Figure 10:**
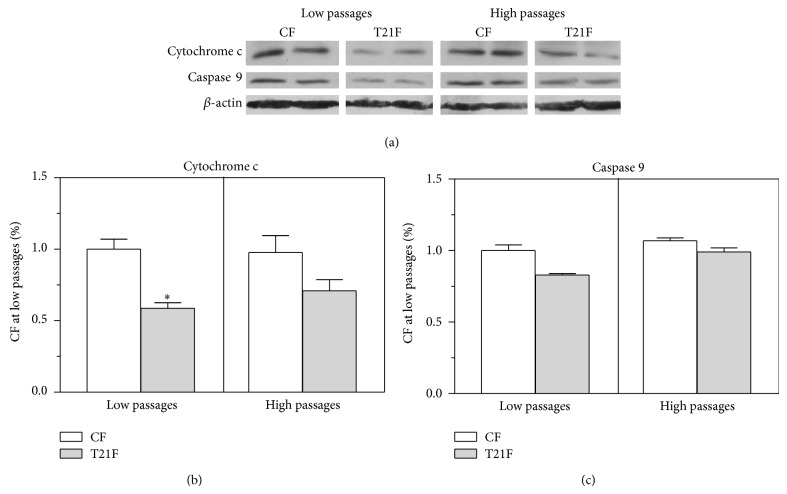
Effect of cell passages on cytochrome c and caspase 9 expression in fibroblasts with trisomy 21 (T21F) (*n* = 5) and controls (CF) (*n* = 5). (a) Representative Western blot of cytochrome c and caspase 9 at low and high passages. (b and c) Protein expression was calculated from optical densities of the bands, measured by an imaging technique and normalized by *β*-actin. Results were normalized to the band intensities measured in untreated fibroblasts at low passages and expressed as mean ± SEM. Statistical differences were analyzed by Student's *t*-test: ^*^ *P* < 0.05.

Since T21F overproduced ROS, and it is known that ROS can also induce apoptosis, cytochrome c and caspase 9 expressions were analyzed by Western blot to ascertain whether apoptosis was activated in T21F fibroblasts (Figure 10). Cytochrome c mass was found to be decreased in T21F both at LP (41%, *P* < 0.05) and at HP (27%) with respect to CF. Cytochrome c mass remained unchanged in control fibroblasts from LP to HP, whereas there was a nonsignificant increase of 15% in T21F. No significant differences were found in caspase 9 expression between both types of cells and between passages, although expression was slightly decreased in T21F with respect to control fibroblasts.

## 4. Discussion

In this study, based on the hypothesis that fibroblasts with trisomy of HSA21 from human fetuses, due to a gene dosage effect, would have increased SOD1 expression, with a subsequent imbalance in enzymatic antioxidant system, we sought to delve into the consequences of such an imbalance which would involve increased ROS production, causing oxidative molecular damage possibly associated with premature cellular senescence. Indeed, the main objectives of this work were to contribute to understanding of the pathophysiologic mechanisms linking cellular metabolism with premature aging and age-related pathologies in DS.

We analyzed the degree of oxidative stress via intracellular ROS levels which were elevated at baseline in T21F, thereby generating chronic oxidative stress; furthermore, the ROS production was always higher in prooxidant treated cells, whether at low or high passages. Although we cannot explain why all cells produced less ROS at higher passages, it may have been due to an increase in the expression of antioxidant enzymes. The fluorescent probe CM-H_2_DCFDA is often considered to detect H_2_O_2_ levels, though it actually detects cellular peroxides and not any specific free radical, since sensitivity of the probe varies according to the reactive species to which it binds; thus, the CM-H_2_DCFDA assay can be considered to quantify oxidative stress [27]. As a consequence of this continuous oxidative stress, cells may suffer molecular oxidative damage since these highly reactive species readily interact with macromolecules, triggering a chain of peroxidation affecting cellular membranes and organelles. Lipids are the molecules most prone to major oxidative injury via a process known as lipoperoxidation which affects structures rich in polyunsaturated fatty acids such as cell membranes, the susceptibility of which increases as the number of double bonds rises and poses a constant threat to cell integrity and function with ensuing tissue damage. In the present study, MDA, the main end-product of LPx, was found to be raised significantly only in T21F cells as replicative senescence progressed. In fact, LPx is an ongoing process which, in normal physiology, acts as a renovator of biological membranes although its excessive activation is linked with the pathogenesis of many diseases and pathological processes; thus, LPx is considered a major factor in the ageing of aerobic cells [28].

Oxidative damage to proteins is one of the modifications leading to severe failure of biological functions and cell death; protein carbonyl groups constitute the most important biomarker of protein oxidation and, as they are more stable than MDA, their determination provides an additional advantage. In this study, carbonylated protein levels were detected and quantified and a significant increase in T21F was observed compared with control cells. Furthermore, carbonylated proteins increased in both cell types as replicative senescence progressed; moreover, a significant increase in oxidative injury to proteins was observed in fibroblasts from DS. This noteworthy novel finding would point to a deleterious role of oxidatively modified proteins in early cell senescence in T21F, thereby linking protein oxidation to premature aging in DS.

Replicative senescence of human fibroblasts has been widely studied and is considered to be a valid model for studying aging. Furthermore, an accumulation of oxidized proteins has been documented in senescent fibroblasts [29]. The age-associated accumulation of oxidized proteins and lipids has been shown to disturb several normal cellular functions, thereby contributing to the aging process by inhibiting the proteasome, an important proteolytic system underlying age-dependent changes [30]. A considerable body of evidence supports the involvement of free radicals in the aging process and an accumulation of cellular components oxidatively damaged with age has been reported, providing evidence that oxidant damage is one of several factors contributing to the aging process [31]. The presence of oxidative stress has long been associated with DS and may be implicated in the development of chronic DS-related health complications [8, 32]; indeed, OS has been shown to occur in the pathogenesis and progression of DS due to a dysregulation of gene/protein expression associated with the trisomy characteristic of DS [33]. The effects of OS in DS from embryonic life onwards have been documented; a study that evaluated OS biomarkers in the amniotic fluid of women carrying DS fetuses indicated that oxidative damage is an early event in DS pathogenesis and might contribute to the generation of deleterious DS phenotypes and abnormal development [34]. Indeed, neurons of DS patients have been found to exhibit a sharp increase in intracellular ROS accompanied by elevated levels of LPx products [35]. The occurrence of chronic oxidative injury in the brain as a risk factor for subsequent neurodegeneration in aged DS subjects had been reported [36]. In other neurodegenerative diseases, and even during aging, disturbances are produced in the intracellular pathways of oxidized protein degradation, leading to a progressive accumulation of oxidatively modified proteins with ensuing loss of cell functions, particularly with advanced age [37]. Through SA-*β*-Gal assay, our results showed cell aging to be more pronounced in DS embryonic fibroblasts, possibly related to the increased ROS and subsequent molecular and cellular oxidative damage.

Oxidative stress and mitochondrial dysfunction are prominent features of DS and since mitochondria are the main source of ROS production, their role is essential in age-related oxidative damage [15]. Trisomy of HSA21 has also been associated with mitochondrial dysfunction in cells and tissues from DS subjects, which has led to the concept that mitochondrial dysfunction contributes to the DS phenotype that includes premature aging [35, 38]. In the present study, basal cellular ROS production at LP was found to be 29% higher in T21F than in CF while, in replicatively aged DS fibroblasts, it was 48% higher than in normal fibroblasts, thus indicating a significant rise in oxidative stress during replicative senescence not reported previously which, from a broader view point, would support the accelerated senescence phenotype characteristic of Down's syndrome. Increased ROS production has also been found to be accompanied by mitochondrial dysfunction, which occurs in DS cells as early as in embryonic life [34]. A study in DS fibroblasts found a selective deficiency of complex I which contributes to mitochondrial ROS overproduction, which further suggests that mitochondrial biogenesis is upregulated in DS [39]. The relationship between oxidative stress and mitochondrial dysfunction is of special importance given the pivotal role of mitochondria not only as a major site of production but also as a target of ROS. Mitochondria constitute the organelle where the ATP is generated and the continuous ROS production could give rise to a drop in ATP synthesis, a process that also occurs during cell aging. We found intracellular ATP content to be significantly decreased in T21F, which could contribute to premature cell aging as observed in DS fibroblasts of the present study. Mitochondrial function could be partially compromised in T21F since they exhibited increased production of ROS, particularly the mitochondrial radical superoxide, and reduced ATP levels. The involvement of possible abnormalities in mitochondrial energy metabolism in DS pathogenesis has been studied, and a strong impairment of mitochondrial ATP synthesis due to a reduction in the catalytic efficiency of certain proteins involved in mitochondrial ATP synthesis has been found [40]. Although a significant decline in intracellular ATP content and increased mitochondrial ROS production were detected in T21F, which together would point to an impaired mitochondrial function, a direct relationship could not be established as this study lacked direct measurements of mitochondrial function.

Most Down's syndrome phenotypes would appear to be related to alterations in gene expression derived from the extra copy of HSA21 and, in line with the “gene dosage effect” hypothesis, some DS features could result from the dosage imbalance of HSA21 genes [41, 42]. Considering that changes in gene expression and protein mass result in alterations in the necessary synergic activity of the antioxidant enzymatic system, the present study aimed to ascertain whether an antioxidant enzyme imbalance existed and, if so, whether it persisted in successive passages. Overexpression of some genes encoded by HSA21 which include SOD1, the most potential inducer of OS since it plays a key role in the antioxidant defense system by catalyzing the dismutation of superoxide radicals, can disturb the activity ratio between SOD and GPx plus CAT, giving rise to a prooxidant status in trisomic cells that may be of key importance in the pathogenesis of DS. In this study, a marked increase in the mRNA of SOD1 and significant increments in protein mass and activity were found in T21F. In a very recent study, Gimeno et al. [43] also found mRNA and protein levels of SOD1 to be increased in DS fibroblasts. However, a point of interest of the present work is that we studied the antioxidant enzyme system during cell replicative senescence in our DS cell model and found SOD1 activity and protein levels to be increased particularly in older trisomic cells. In living organisms, trisomic cells tend to compensate for the excessive production of H_2_O_2_ by increasing the enzymes that inactivate it. However, in this* in vitro* study, decreases (albeit with no significant differences) in expression and activities of GPx and CAT were observed; thus, the SOD/CAT+GPx activity ratio was significantly increased in T21F and the imbalance was more pronounced in aged cells. The SOD/GPx, SOD/CAT, and SOD/(GPx+CAT) ratios were higher in T21F than in CF at low passages and this interenzymatic activity misbalance was accentuated with replicative senescence. To our knowledge, these specific differences in the antioxidant enzyme defense system in relation to cell senescence have not been described previously. As a result of increased SOD1 activity without the concomitant increase in complementary antioxidant defense mechanisms, an excessive production of endogenous H_2_O_2_ is generated, thereby oxidizing biomolecules such as proteins and lipids and damaging important cellular components, as we observed in this study.* In vivo*, the overproduction of H_2_O_2_ through the action of SOD1 has long been known to be the main source of ROS in DS patients [8, 32, 44, 45], powered by an imbalance in SOD/(GPx+CAT) ratio activities, thereby inducing systemic oxidative stress [46] which appears to be a fundamental factor contributing to a senescent phenotype characteristic of DS. However, the free radical theory of aging is not the only theory proposed to explain the mechanism(s) involved in aging at molecular and cellular levels since other mechanisms such as telomere senescence, genomic instability, and the mitochondrial hypothesis of aging also participate in cell senescence [47, 48]. To gain further insight into the mechanisms that might be implicated in the early loss of proliferative capacity and growth of T21F with replicative senescence, the expression of p21 and p53 proteins was studied in fibroblasts with trisomy 21 during the cell aging process* in vitro*.

This study found that DS fibroblasts entered a state of premature senescence as shown by SA-*β*-Gal assay and p21 assessment; it has been reported that, in human fibroblasts, protein p21 levels rise through passages of primary cells [49] and that the overexpression of p21 leads to cell cycle arrest and the appearance of cellular senescence markers such as SA-*β*-Gal activation, cell hypertrophy and flattening [50], and enzymatic and morphologic findings, respectively, of this study on embryonic DS cells. The higher oxidative stress levels seen in DS fibroblasts in this work could increase the expression and activation of p53, one of the main effectors of p21 [51] and, interestingly, many of the neurodegenerative and immune defects of DS patients correlate with an increased apoptosis rate linked to an increased expression of the proapoptotic tumour suppressor p53 [52–54]. However, neither p53 nor the proapoptotic proteins cytochrome c and caspase 9 were altered in our DS cells, although it should be noted that Western blots were performed with total cell lysates and thus it was not possible to ascertain whether cytochrome c was being released from the mitochondria.

While some reports have pointed to the activation of the apoptotic pathway as a pathophysiological mechanism behind DS phenotypes, the opposite might also be true. Under certain conditions, p21 functions as a potent antiapoptotic factor acting at different levels of the cell death cascade. In fact, p21 can promote resistance to apoptosis by inhibiting activation of caspase 9 and cytochrome c release from mitochondria [51]. Moreover, although altered apoptosis has been suggested as one of the mechanisms responsible for different DS phenotypes, most* in vivo* studies have failed to find alterations in this process [55]. For instance, it has recently been reported that overexpression of Dyrk1a, a member of the tyrosine-phosphorylation-regulated protein kinase family located at chromosome 21 [56], reduces the magnitude of physiological apoptosis in the developing retina of trisomic Ts65Dn mice, leading to a phenotype that resembles that seen in DS individuals [57]. Therefore, Dyrk1a overexpression in DS fibroblasts could account for the diminished levels of caspase 9 and apoptosis seen in these cells. However, as Liao and colleagues pointed out [58], the story might not be this simple since some Down syndrome-associated proteins are also found to induce apoptosis in cells. The results herein show that senescence, but not apoptosis, appears prematurely in cultured DS fibroblasts, which could account for the appearance of premature ageing signs seen in individuals with Down's syndrome.

## Figures and Tables

**Table 1:** Effect of prooxidants on cell viability.

Treatment	Low passages		High passages	
	CF	T21F	CF	T21F
Untreated	100.0 ± 3%	100.0 ± 3%	100.0 ± 3%	100.0 ± 2%
AAPH	97.8 ± 2%	99.1 ± 3%	98.0 ± 3%	95.1 ± 2%
TBHP	93.0 ± 2%	93.7 ± 2%	96.1 ± 2%	89.0 ± 2%^a^

Control (*n* = 5) and trisomy 21 (*n* = 5) fibroblasts were grown in 96-well microplates and incubated for one hour with Hank's buffered salt solution (HBSS) (untreated) or supplemented with 10 mM AAPH or 100 *μ*M TBHP. Cell viability was determined by XTT assay and expressed as a percentage of XTT reduction compared with untreated fibroblasts. Values are expressed as mean ± SEM of six separate experiments performed in quadruplicate. Statistical differences between low- and high-passage groups were analyzed by Student's *t*-test; ^a^ *P* < 0.01.

**Table 2:** Effect of replicative aging on lysosomal hydrolases.

	Low passages			High passages		
	*β*-Gal (pH 6)	*β*-Gal (pH 4)	*β*-Gluc	*β*-Gal (pH 6)	*β*-Gal (pH 4)	*β*-Gluc
CF	6.30 ± 0.4	24.88 ± 4.6	4.22 ± 0.5	7.97 ± 0.7	23.42 ± 2.2	6.54 ± 0.7^a^
T21F	7.42 ± 0.9	23.68 ± 2.3	4.53 ± 0.7	8.19 ± 0.6	21.05 ± 2.0	5.96 ± 0.7

Activities of lysosomal enzymes, *β*-galactosidase, and *β*-glucuronidase in cell homogenates of control (*n* = 5) and trisomy 21 (*n* = 5) fibroblasts at low and high passages. Activities were corrected by the protein content of the cellular homogenates. The *β*-galactosidase activity was determined at pH 4.0 and 6.0. Results are expressed as mean ± SEM. Statistical differences between passages were analyzed with Student's *t*-test: ^a^ *P* < 0.05.

## Abbreviations

AAPH: 2,2′-Azobis-2-methyl-propanimidamide, dihydrochloride
DS: Down's syndrome
HP: High passages
LP: Low passages
LPx: Lipoperoxidation
OS: Oxidative stress
PCG: Protein carbonyl groups
PD: Population doublings
ROS: Reactive oxygen species
T21F: Fibroblasts with trisomy 21
TBHP: * tert*-Butyl hydroperoxide.
